# Theoretical predicted high-thermal-conductivity cubic Si_3_N_4_ and Ge_3_N_4_: promising substrate materials for high-power electronic devices

**DOI:** 10.1038/s41598-018-32739-x

**Published:** 2018-09-26

**Authors:** Huimin Xiang, Zhihai Feng, Zhongping Li, Yanchun Zhou

**Affiliations:** Science and Technology on Advanced Functional Composite Laboratory, Aerospace Research Institute of Materials and Processing Technology, No. 1 South Dahongmen Road, Beijing, 100076 China

## Abstract

Ceramic substrates play key roles in power electronic device technology through dissipating heat, wherein both high thermal conductivity and mechanical strength are required. The increased power of new devices has led to the replacement of Al_2_O_3_ by high thermal conducting AlN and further β-Si_3_N_4_ based substrates. However, the low mechanical strength and/or anisotropic mechanical/thermal properties are still the bottlenecks for the practical applications of these materials in high power electronic devices. Herein, using a combination of density functional theory and modified Debye-Callaway model, two new promising substrate materials γ-Si_3_N_4_ and γ-Ge_3_N_4_ are predicted. Our results demonstrate for the first time that both compounds exhibit higher room temperature thermal conductivity but less anisotropy in expansion and heat conduction compared to β-Si_3_N_4_. The mechanism underpins the high RT κ is identified as relatively small anharmonicity, high phonon velocity and frequency. The suitability of these two nitrides as substrate materials was also discussed.

## Introduction

The growing consumption of energy and emission of greenhouse gas have great impact on our society. To solve the environmental issues that we are facing, gradually reconstructing the energy sources from fossil fuel to electric power is the general direction. To achieve such a goal, higher voltage, larger current, greater power density and smaller size has become the main trend in power electronic device technology. However, large amounts of Joule heat will be produced in power electronic devices when the current is conducted. Thus, the normal functionality of devices depends on the dissipation of heat through insulating ceramic substrates^1^. There is thereby an urgent need but it is still a significant challenge to rationally design and fabricate high thermal conducting and reliable substrate materials.

To realize the functions of electrical insulation, heat dissipation, and semiconductors’ supporting, both high thermal conductivity and good mechanical reliability are required to ceramic substrates. Due to the high thermal conductivity and low thermal expansion close to that of the semiconductor silicon^2,3^, AlN has been selected to replace Al_2_O_3_ for higher power devices. The low mechanical strength of AlN, however, has caused cracking under thermal stresses^4^, which will lead to the failure of the devices during service. Thus, extensive efforts have been dedicated to exploring and developing new high strength and high thermal conductivity ceramic materials. β-Si_3_N_4_ has high theoretical thermal conductivity of over 250 W∙m^−1^∙K^−1^ and has been considered as an alternate ceramic substrate for AlN^5,6^. Experimentally, high thermal conductivity κ of 177 W∙m^−1^∙K^−1^ has been reported for reaction bonded and post sintered silicon nitride with proper control of the lattice oxygen contents^7^. The foregoing result has engendered an enormous amount of work on developing high κ β-Si_3_N_4_ for high power electrical devices. However, the anisotropic thermal and mechanical properties resulted from the structure characteristic of β-Si_3_N_4_ is one of the main issues limiting the practical applications of β-Si_3_N_4_ based substrate material. For example, in thermal cycles, anisotropy in thermal expansion and conduction expedites the accumulation of thermal stress, degrades the mechanical properties and eventually results in the failure of substrates. Therefore, search for new ceramic materials with better thermal and mechanical properties is eagerly awaited for the development of high power electronic devices.

γ-Si_3_N_4_, the third polymorph of silicon nitride, crystallizes in spinel structure and is stable at ambient temperature and pressure^8,9^. This new form of silicon nitride has attracted much attention since its discovery due to its unique crystal structure, large direct band gap (4.8 eV)^10^, high hardness (35 GPa)^11–13^, high toughness (3.5 MPa∙m^1/2^)^13^. Intriguingly, mechanical properties of γ-Si_3_N_4_ such as elastic moduli and hardness are superior to those of β-Si_3_N_4_^6,13^. It is generally accepted that the high κ materials have simple crystal structure and constituting low average atomic mass with strong interatomic bonding^14^. Thus, γ-Si_3_N_4_ is anticipated to prevail in thermal conductivity. In addition, γ-Si_3_N_4_ is particularly attractive due to the isotropy in thermal expansion and conductivity endowed by its high structure symmetry, which makes it promising as an alternative ceramic substrate material in high power electronic devices.

Unfortunately, the thermal conductivity of γ-Si_3_N_4_ has rarely been investigated. Using a simple Slack’s model, Morelli *et al*. reported a κ value of 80 W∙m^−1^∙K^−1^ at room temperature^5^. This relatively small value contradicts with our expectation and the detail information about the thermal transportation has not been revealed. To gauge the suitability of using γ-Si_3_N_4_ as a new ceramic substrate in high power electronic devices, in this contribution, we attempt to predict the thermal transport property of this potential high thermal conductivity material and its analogue γ-Ge_3_N_4_, and link the heat conduction properties of these two nitrides to their structure characteristic. With the help of first principles calculations and modified Debye-Callaway model, the high thermal conductivity at room temperature of both nitrides is confirmed and the suitability of both compounds as substrate materials will be discussed.

## Results

### Structure and elastic constants

**Figure 1:**
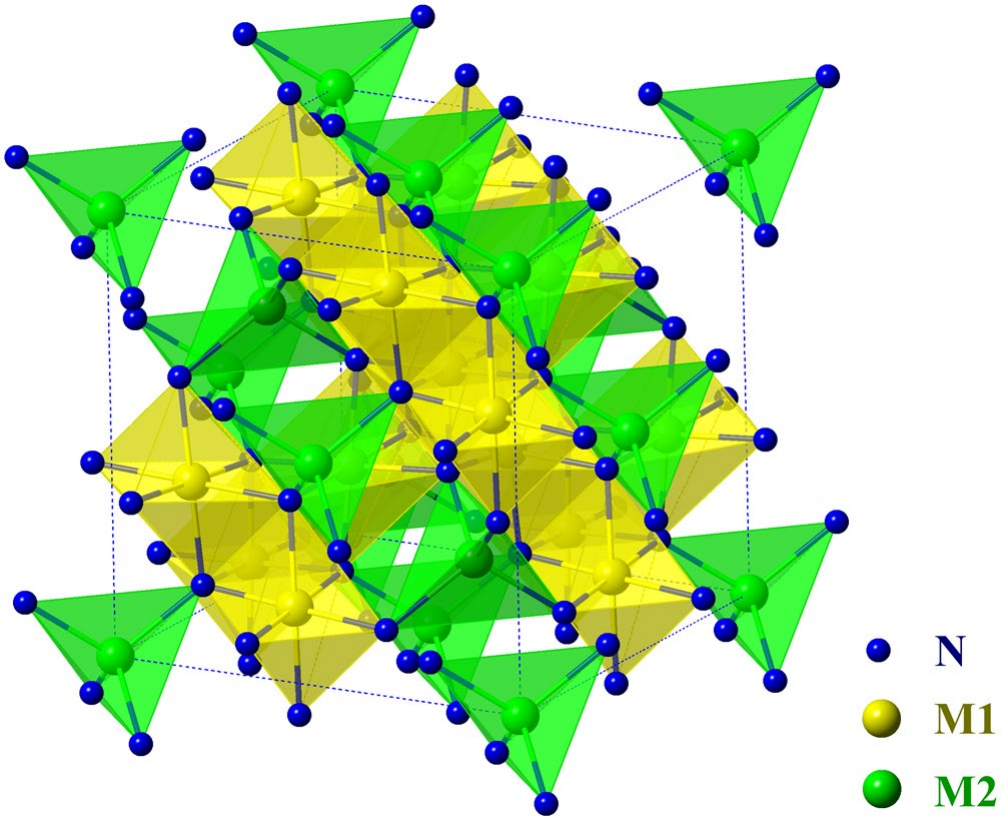
Crystal structure of γ-M_3_N_4_ (M = Si, Ge). The blue balls represent the nitrogen atoms, the yellow balls are octahedrally coordinated metal atoms, and the green balls are fourfold coordinated metal atoms.

γ-M_3_N_4_ (M = Si, Ge), also known as c-M_3_N_4_, crystallize in spinel structure with a space group of $F{\rm{d}}\bar{3}m$. The structure is illustrated in Fig. 1. The Bravais lattice of an ideal spinel structure consists of a fcc sublattice of N with metal atoms occupying one eighth of the interstitial tetrahedral sites and one half of the octahedral sites^12^. For convenience, the octahedrally coordinated metal atoms are denoted as M1, and the fourfold coordinated ones are labeled as M2. The theoretical lattice parameter, *a*, is listed in Table 1, together with reported experimental and theoretical works for comparison^13,15,16^. The deviation between our work and previous reported results on the structure parameter is less than 1% for both nitrides.

**Table 1:** Ground-state lattice constants (Å), second-order elastic coefficients (GPa), bulk modulus *B* (GPa), shear modulus *G* (GPa) and Young’s modulus *E* (GPa) of γ-Si_3_N_4_ and γ-Ge_3_N_4_.

	*a*	*c*_11_	*c*_12_	*c*_44_	*B*	*G*	*G/B*	*E*	Reference
γ-Si_3_N_4_	7.751	530	186	330	301	254	0.844	595	This work
	7.742				308				^15^
	7.737				303	248		584	^13^
	7.697	533	191	341	305	258			^15^
	7.789				285				^16^
γ-Ge_3_N_4_	8.267	387	151	224	230	173	0.752	415	This work
	8.300								^17^
	8.213	395	165	235	242	176			^15^
	8.288	368	146	223	220	169	0.769	403	^18^

The second-order elastic constants and mechanical moduli of γ-M_3_N_4_ are obtained from DFT calculations and listed in Table 1. The data from previous works are also included^15,17,18^. The elastic constants and mechanical properties of these two nitrides are slightly smaller than the reported experimental and theoretical results due to the overestimated lattice parameter where GGA is implemented in the calculations. Overall, the agreement between our theoretical results and experimental data are quite satisfactory. The mechanical moduli of Si_3_N_4_ are considerably larger than those of Ge_3_N_4_, indicating stronger bonding strength of Si_3_N_4_.

### Phonon dispersion and mode-Grüneisen parameters

**Figure 2:**
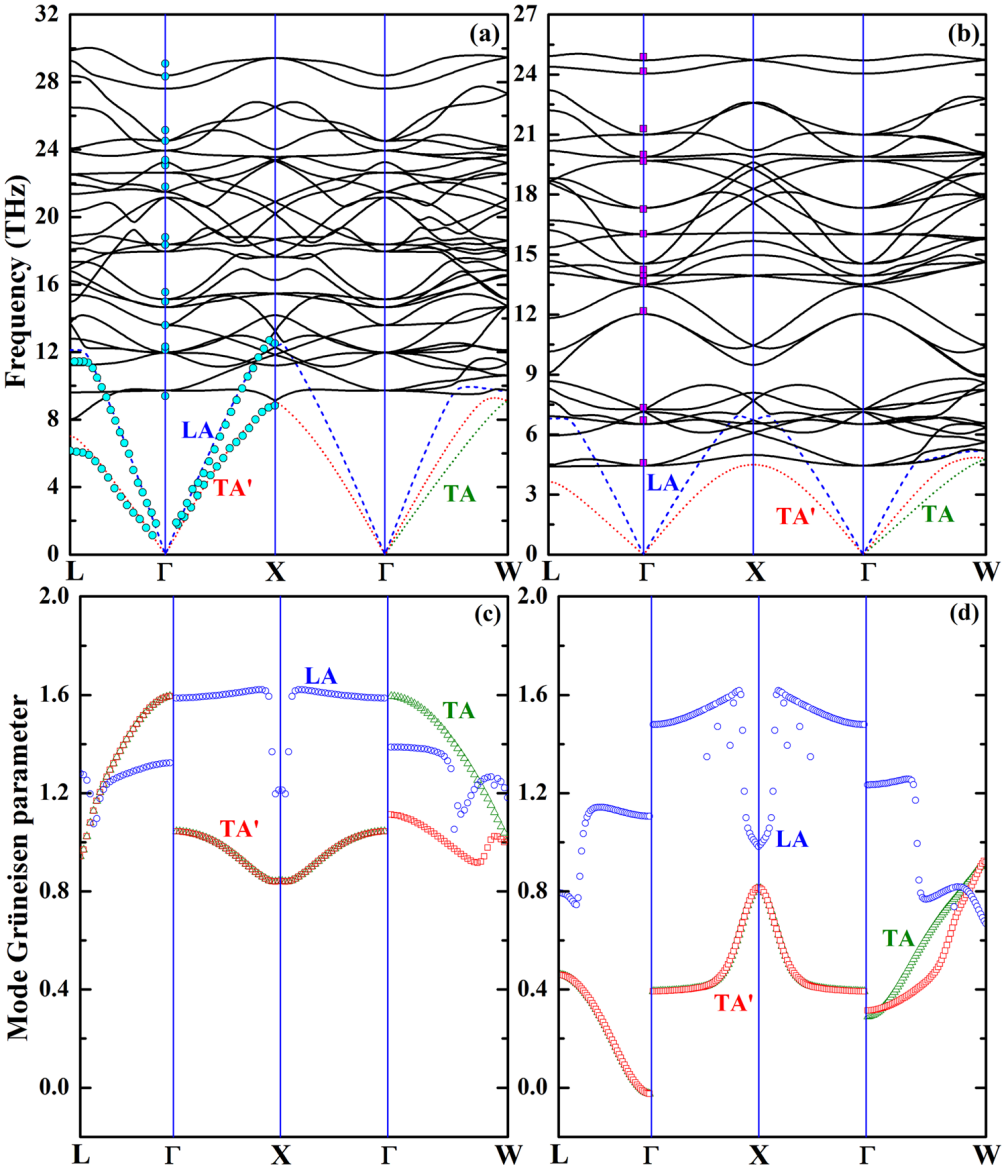
Phonon dispersion and mode Grüneisen parameters of (**a**,**c**) γ-Si_3_N_4_ and (**b**,**d**) γ-Ge_3_N_4_. The theoretical results from previous reports are also included for comparison. The cyan circles are from ref.^19^, while the magenta squares are from ref.^20^.

Phonon dispersion curves along high-symmetry directions in the BZ are calculated for γ-Si_3_N_4_ and γ-Ge_3_N_4,_ as shown in Fig. 2(a,b). For both nitrides, the profiles of the dispersions are in general similar; no obvious gap between the acoustic and low-lying optical branches exists. The main difference is that the frequencies of the phonons of γ-Si_3_N_4_ are fairly larger than those of γ-Ge_3_N_4_, which reconfirms larger force constants of chemical bonds in γ-Si_3_N_4_. The validation of our calculation on the phonon dispersions of these two nitrides is conducted by including the previous reported dispersion^19,20^, as shown in Fig. 2(a,b). The absence of acoustic-optical branches gap is an indication of possible acoustic-optical interaction, especially for longitudinal acoustic (LA) modes of both nitrides. Such interaction is the main source that shortens the lifetime of acoustic modes when the temperature is high enough to excite the low-lying optical modes. Another interesting phenomenon in the dispersion curves of both compounds is the avoided crossing between LA and low-lying optical modes at the BZ boundary^21^, which leads to abrupt changes in the slope of LA mode and the calculated mode Grüneisen parameters γ_*i*,*q*_, as shown in Fig. 2. The curving back of γ-Si_3_N_4_ is more pronounced than γ-Ge_3_N_4_. While for transverse acoustic (TA) modes, no such avoided crossings are observed, and the normal feature is maintained with the frequencies increasing linearly toward the BZ boundary, which implies that the interaction between TA modes and low-lying optical modes is limited, and the scattering of TA modes is weaker than that of LA mode.

The thermal conductivity of an acoustic mode is determined by the mode relaxation time, which is closely related to the mode Grüneisen parameter γ_*i*_, as illustrated in Eq. (5). Therefore, the mode Grüneisen parameter is essential to understand the feature of thermal transport. Using Eq. (6), we calculated the mode Grüneisen parameter for all acoustic modes, as shown in Fig. 2(c,d). Throughout the BZ, the mode Grüneisen parameters of these two compounds are positive besides several ***q*** around Γ point along Γ-L high symmetry direction of γ-Ge_3_N_4_. As stated above, the avoided crossings in the phonon dispersion are only perceived between LA and low-lying optical modes, the mode Grüneisen parameters of LA modes are considerably larger than those of TA modes, especially along Γ-X high symmetry direction. This is an indication of severer phonon scattering of LA modes, compare with TA modes. The Grüneisen parameter curves of LA modes for these two nitrides are roughly analogous in profile. For γ-Si_3_N_4_, since the curving back of acoustic modes is more pronounced, the mode Grüneisen parameters are substantially larger, compared with γ-Ge_3_N_4_. Using the dispersion of mode Grüneisen parameters, we calculated the average mode Grüneisen parameters of LA and TA modes by Eq. (7), which are 1.43, 1.10, 1.24 and 1.15, 0.43, 0.46 for γ-Si_3_N_4_ and γ-Ge_3_N_4_, respectively. For cubic Si_3_N_4_, Jiang *et al*. determined γ = 1.23 from thermal expansion^22^, and a value of 1.2 can be deduced from compressibility measurements conducted by He *et al*.^23^. These data is quite consistent with our prediction, which is 1.24 from the average of three acoustic mode Grüneisen parameters. As regard γ-Ge_3_N_4_, no experimental data has been reported. Morelli *et al*. assumed that the Grüneisen parameter of cubic Ge_3_N_4_ is 1.2 in the estimation of thermal conductivity of germanium nitrides^5^. This value is considerably larger than our prediction, which is 0.68. Since the thermal conductivity κ is proportional to γ^−2^, the thermal conductivity of γ-Ge_3_N_4_, 50 W∙m^−1^∙K^−1^ at 300 K predicted by Morelli *et al*.^5^, is highly underestimated.

Using the phonon dispersion and mode Grüneisen parameters, all decisive parameters, e.g. average acoustic group velocity *v*_i_, mode-dependent Debye temperature *θ*_i_ and mode average Grüneisen parameter γ_i_, that required in Eq. (3) and (5) to calculate the phonon relaxation time and thermal conductivity are derived and tabulated in Table 2. For both nitrides, large group velocity, Debye temperature and average Grüneisen parameter of LA modes is observed. The influence of these parameters on the thermal conductivity behavior of acoustic modes will be discussed later.

**Table 2:** The Longitudinal and transverse average group velocities (*v*_i_ in km/s), Debye temperatures (*θ*_i_ in K), and Grüneisen parameter ${\mathop{{\rm{\gamma }}}\limits^{-}}_{i}$ for each acoustic phonons.

	*v*_LA_	*v*_TA/TA'_	*θ*_LA_	*θ*_TA'_	*θ*_TA_	$\mathop{{\rm{\gamma }}}\limits^{-}$_LA_	$\mathop{{\rm{\gamma }}}\limits^{-}$_TA'_	$\mathop{{\rm{\gamma }}}\limits^{-}$_TA_
γ-Si_3_N_4_	11.95	9.04	600	484	434	1.43	1.10	1.24
γ-Ge_3_N_4_	7.82	5.42	333	259	216	1.15	0.43	0.46

### Intrinsic thermal conductivity of γ-M_3_N_4_

**Figure 3:**
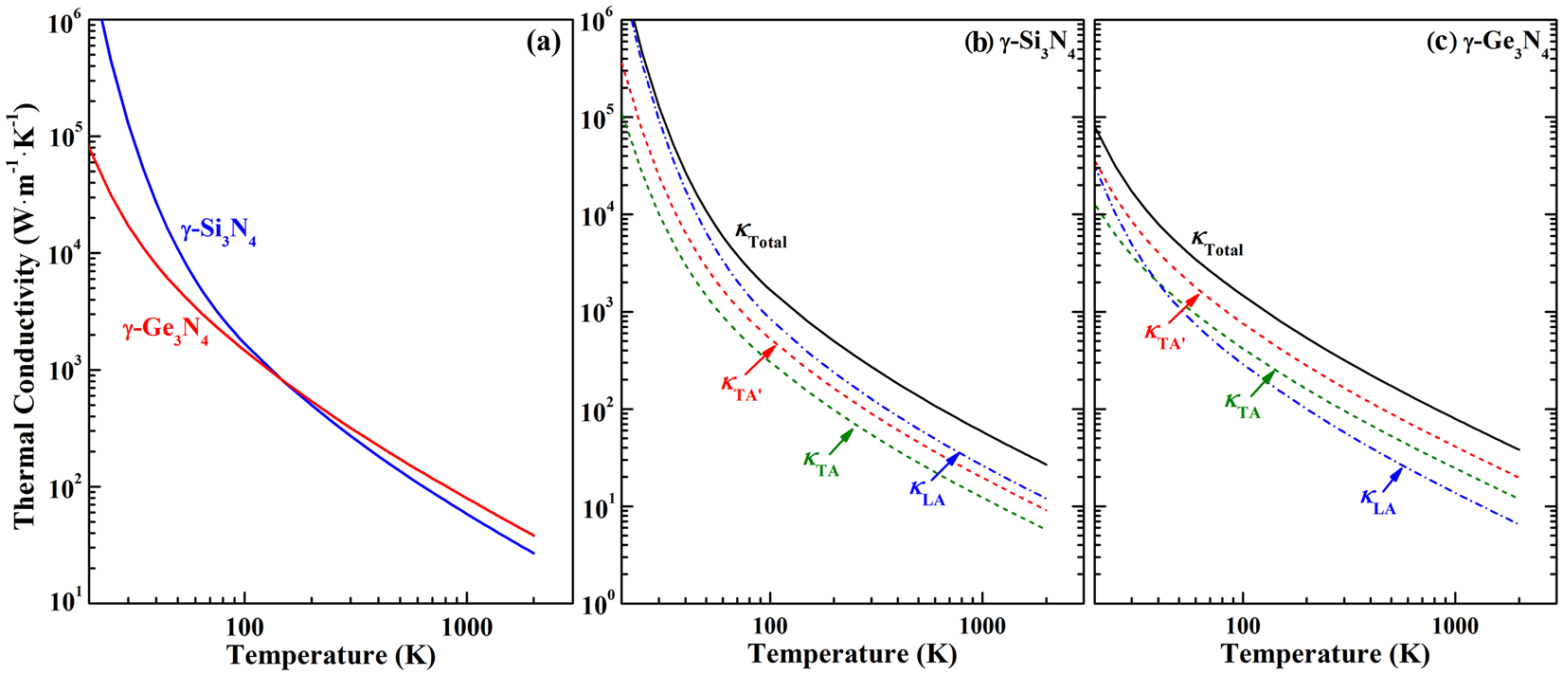
(**a**) Intrinsic lattice thermal conductivity of γ-Si_3_N_4_ and γ-Ge_3_N_4_, and contribution of longitudinal acoustic (LA) phonon (dash dot line) and transverse acoustic (TA) phonon (dash line) to the thermal conductivity of (**b**) γ-Si_3_N_4_ and (c) γ-Ge_3_N_4_.

The temperature-dependent intrinsic thermal conductivity (*κ*_L_) of γ-M_3_N_4_ is calculated by the approach of modified Debye-Callaway model, as shown in Fig. 3(a). The *κ*_L_ of these two nitrides decreases rapidly with the rising of temperature. However, the temperature dependences of total thermal conductivity *κ*_L_ of these two compounds are different. The efficiency of heat transfer in γ-Si_3_N_4_ is overwhelmingly higher than that in γ-Ge_3_N_4_ at low temperatures. With temperature increasing, *κ*_L_ of Si_3_N_4_ decreases faster, and above 300 K, the thermal conductivity of γ-Ge_3_N_4_ exceeds that of γ-Si_3_N_4_. Different temperature-dependency of thermal conductivity is the manifestation of phonon transport mechanism at different temperature ranges. At low temperatures, only the low-energy phonons with short wave vector ***q***, i.e. the phonons adjacent to BZ center, are excited. Without long wave vector, the resistive phonon-phonon (*p-p*) interaction Umklapp process is suppressed due to the requirement of conservation of momentum. Thus, the decisive element that determines the thermal conductivity is the group velocity of these excited BZ-center phonons. As listed in Table 2, considerably large group velocity of γ-Si_3_N_4_ brings about large thermal conductivity at low temperatures. On the contrary, at high temperatures, the Umklapp process governing the *p-p* interaction, the interaction strength, which is quantified by the Grüneisen parameter $\mathop{{\rm{\gamma }}}\limits^{-}$, is the main factor in the calculation of thermal conductivity. Due to the γ^−2^ dependency of thermal conductivity, relatively small $\mathop{{\rm{\gamma }}}\limits^{-}$ is positive to the enhancement of thermal transport of γ-Ge_3_N_4_ at high temperatures.

To gain further insight into the heat transfer feature of these two nitrides, the contributions from different acoustic modes to the total *κ*_L_ are also calculated, as illustrated in Fig. 3(b,c). Of great interest, within studied temperature range, the contribution from LA is larger than those from TA modes for γ-Si_3_N_4_, while for γ-Ge_3_N_4_, TA modes contribute more. The thermal conductivity of LA mode in γ-Ge_3_N_4_ is only 58 W∙m^−1^∙K^−1^ at 300 K, being considerably lower than those from two TA modes, which are 96 and 164 W∙m^−1^∙K^−1^, respectively. Elucidating the different thermal transportation character of LA and TA modes of these two compounds relies on the examination of decisive parameters, i.e. average acoustic group velocity *v*_i_, mode-dependent Debye temperature *θ*_i_ and mode average Grüneisen parameter $\mathop{{{\rm{\gamma }}}_{{\rm{i}}}}\limits^{-}$, in Eq. (5). For γ-Si_3_N_4_, although the Grüneisen parameters of LA mode is 23% and 13% larger than two TA modes, the group velocity and Debye temperature of LA mode is 24% larger than those of TA modes. The influence of *v*_i_ and *θ*_i_ on the thermal conductivity overwhelms the effects of Grüneisen parameter and leads to the larger contributions from LA mode. In contrary, for γ-Ge_3_N_4_, the velocity and Debye temperature of TA modes are just 31% and 29% smaller than those of LA mode are, while γ of TA modes is 63% smaller, which is the decisive ingredient in the determination of thermal transportation property of γ-Ge_3_N_4_.

### Effect of grain size

**Figure 4:**
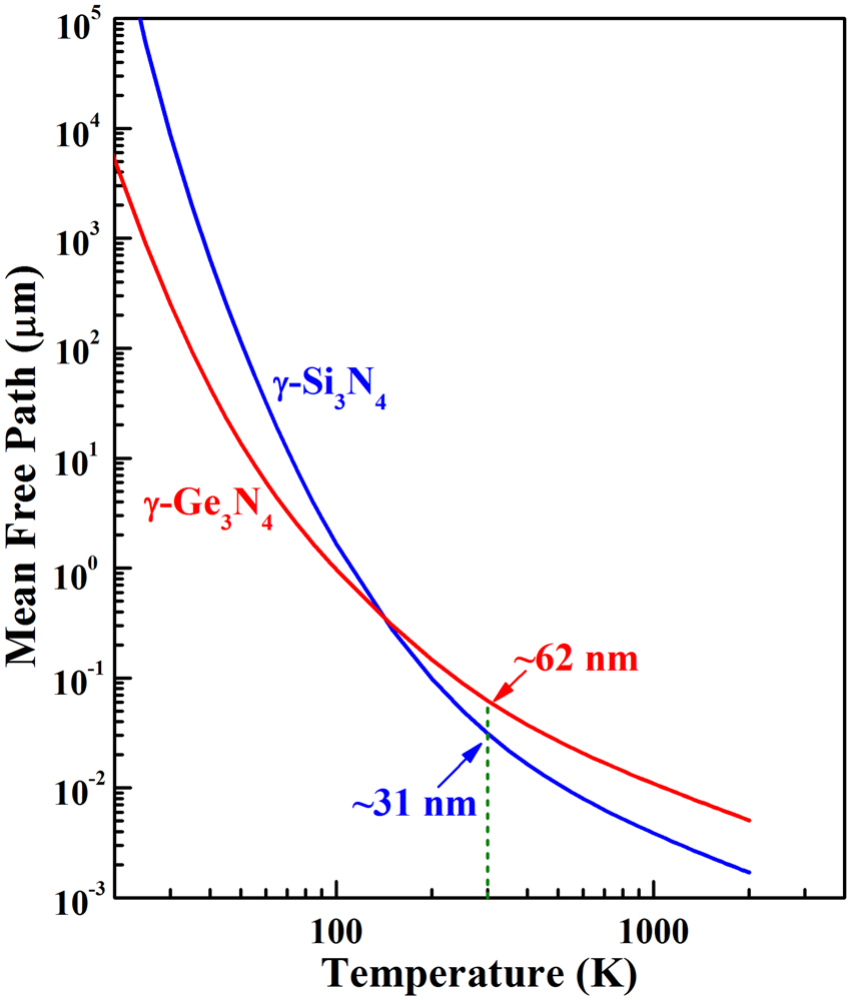
Temperature-dependent phonon mean free path of γ-Si_3_N_4_ and γ-Ge_3_N_4_.

Using the thermal conductivity at different temperatures, we can estimate the phonon mean free path (MFP) of these two nitrides by:

1 $$\kappa =\frac{1}{3}{C}_{v}vl$$

where *C*_*v*_ is the heat capacity per volume, *v* is the velocity of phonons and *l* is the MFP. As shown in Fig. 4, with temperature increasing, the calculated MFP of these two nitrides decreases rapidly from several centimeters to tens of nanometers. The average phonon MFP of γ-Si_3_N_4_ and γ-Ge_3_N_4_ is about 31 nm and 62 nm at 300 K, respectively. These data is significant in tailoring of microstructure and thermal conductivity, since the grain boundary is a main annihilation source for phonons with MFP larger than diameter of the grain. In a typical polycrystalline sample, the grain size ranges from several to hundreds of micrometers. Thus, for these two nitrides, the phonon scattering from the grain boundary below 80 K is much severe because the phonon MFP is larger than the grain size. In order to depict the thermal conductivity at low temperatures correctly, the scattering rate of grain boundary (Eq. (9)) needs to be included in the calculation of total resistive phonon scattering rate. Since the scattering rate of grain boundary is in inverse proportion to the diameter of grain, the effects of two sorts of samples with grain size in 10 μm and 100 μm are considered in our calculations.

**Figure 5:**
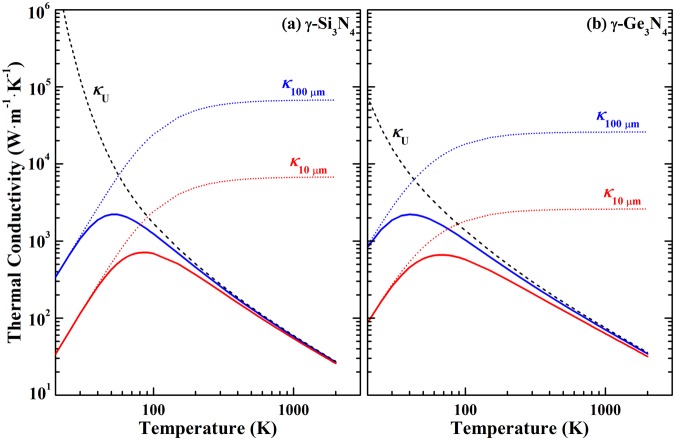
The effect of grain size on the lattice thermal conductivity for (**a**) γ-Si_3_N_4_ and (**b**) γ-Ge_3_N_4_. The dash line is the thermal conductivity without the effect of grain boundary, the dot line is the thermal conductivity with the effect of grain boundary considered.

The temperature-dependent thermal conductivities of samples with grain size of 10 μm and 100 μm for γ-Si_3_N_4_ and γ-Ge_3_N_4_ are shown in Fig. 5. Clearly, a global maximum value exists in the thermal conductivity curve of polycrystalline materials, this is resulted from two competitive phonon scattering mechanisms: *p-p* interaction and grain boundary scattering. At low temperatures, size effect due to the boundary scattering dominates and endows the thermal conductivity with *T*^3^ dependency. At high temperatures, however, conductivity due to boundary scattering approaches constant when all acoustic phonons are excited, and the effect of *p-p* interaction overwhelms the size effect, resulting in *T*^−1^ dependency of thermal conductivity at high temperatures. Apparently, the maximum thermal conductivities for these two nitrides with different grain sizes differ in value and temperature. Global maximum shifts to lower temperatures for larger grain size materials. For instant, the largest thermal conductivity of γ-Si_3_N_4_ with a grain size of 100 μm is 2161 W∙m^−1^∙K^−1^ at 50 K, three times larger than that of sample with a grain size of 10 μm, which is 695 W∙m^−1^∙K^−1^ at 85 K. For γ-Ge_3_N_4_, these two values are 2284 W∙m^−1^∙K^−1^ at 45 K and 682 W∙m^−1^∙K^−1^ at 70 K. respectively. Since the *p-p* interaction dominates, the thermal conductivity of polycrystalline samples of these two nitrides is free of grain size above room temperature (RT). As listed in Table 3, the RT conductivity for γ-Si_3_N_4_ and γ-Ge_3_N_4_ is approximately 300 W∙m^−1^∙K^−1^, which is comparable to the theoretical limits of β-Si_3_N_4_^5,6^. Such large RT thermal conductivities promise these two nitrides potential applications in electronic devices.

**Table 3:** The Grüneisen parameter $\mathop{{\rm{\gamma }}}\limits^{-}$, and thermal conductivity (*κ* in W∙m^−1^∙K^−1^) of three samples with different grain size of γ-Si_3_N_4_ and γ-Ge_3_N_4_ at RT (300 K) and 1400 K.

	$\mathop{{\rm{\gamma }}}\limits^{-}$	*κ*_RT_	*κ*_1400K_
γ-Si_3_N_4_	1.24	272 (single crystal)	40 (single crystal)
		254 (100 μm)	39 (100 μm)
		220 (10 μm)	37 (10 μm)
γ-Ge_3_N_4_	0.68	318 (single crystal)	55 (single crystal)
		284 (100 μm)	53 (100 μm)
		222 (10 μm)	48 (10 μm)

## Discussion

The commonly accepted criteria^14^ in searching for materials with high κ include (i) simple crystal structure, (ii) low average atomic mass, *M*_av_, (iii) strong interatomic bonding, and (iv) low anharmonicity. Thus, compounds with light constituting elements and high mechanical moduli and/or hardness are generally considered as potential candidates with high κ, such as diamond^24^, cubic-BN^25^, and SiC^26^. For γ-Si_3_N_4_ and γ-Ge_3_N_4_, they are constituted by light elements, crystallize in simple spinel structure, and γ-Si_3_N_4_ is considered as one of the third hardest materials next to diamond and *c*-BN^13^. Following these guidelines, it is quite natural to presume that cubic Si_3_N_4_ and Ge_3_N_4_ are good thermal conductors. Since the elastic moduli are higher and the average atomic mass is lower than those of γ-Ge_3_N_4_, γ-Si_3_N_4_ is expected to exhibit higher thermal conductivity. In our predictions, the high thermal conductivities of these two nitrides are verified, *κ* at RT is higher than 250 W∙m^−1^∙K^−1^ for samples with grain size of 100 μm for both compounds. However, the thermal conductivity of Ge_3_N_4_ is as high as that of Si_3_N_4_, which is contrary to our expectations. The mechanism lies behind unexpected high κ of Ge_3_N_4_ is that rather small Grüneisen parameter compensates the negative effects of low group velocities and Debye temperature on the thermal conductivity. The origin of small $\mathop{{\rm{\gamma }}}\limits^{-}$ is the existence of gap between low-lying optical modes and TA modes in Ge_3_N_4_, which vanishes in Si_3_N_4_, as shown in Fig. 2. The acoustic-optic frequency gap freezes out scattering process involving low-frequency TA phonons due to the restriction of phonon energy and momentum conservation. Morelli *et al*. reported a predicted RT κ of 50 W∙m^−1^∙K^−1^ for γ-Ge_3_N_4_ with an assumed average Grüneisen parameter $\mathop{{\rm{\gamma }}}\limits^{-}$ = 1.2^5^. This value is considerably lower than our prediction; large discrepancy is the result of inaccuracy $\mathop{{\rm{\gamma }}}\limits^{-}$ used in the calculation of thermal conductivity. Regarding γ-Si_3_N_4_, a divergence in thermal conductivity between our prediction and the result provided by Morelli *et al*. is found despite of similar Grüneisen parameter implemented in the calculation^5^. Relatively small acoustic mode Debye temperature employed in their calculation is the main reason for the low predicted RT κ of γ-Si_3_N_4_.

γ-Si_3_N_4_ shares the same constitution elements and average atomic mass with β-Si_3_N_4_, a promising material for high-power device with theoretical κ higher than 250 W∙m^−1^∙K^−1^ and verified by experiments in some extents^5–7^. However, γ-Si_3_N_4_ does not prevail against β-Si_3_N_4_ in thermal conductivity despite γ-Si_3_N_4_ is higher in elastic moduli and hardness^6,13^. The determinative element is the anharmonicity of the structure, which is quantified by Grüneisen parameter. The $\mathop{{\rm{\gamma }}}\limits^{-}$ of γ-Si_3_N_4_ is nearly twice as large as that of β-Si_3_N_4_^5^. Examining the structures of these two nitrides, it is noticeable that the main difference between these two phases is the building blocks. The structure of β-Si_3_N_4_ can be described as stacking of Si-N tetrahedra in three dimensions by sharing ntrigen atom^6^. While for γ-Si_3_N_4_, there are two types of Si-N polyhedra, i.e. Si-N tetrahedron and Si-N octahedron. The bond length and strength of two types of Si-N bonds are diverse, Si_oct_-N (1.882 Å) is longer and weaker than Si_tet_-N (1.777 Å). Diversity in bonding strength is a barrier to the transportation of phonons, which enhances the scattering of phonons and increases the heat resistance.

The promising perspective of γ-Si_3_N_4_ and γ-Ge_3_N_4_ as ceramic substrates for high-power electronic devices is defined not only by their high thermal conductivity but also by their chemical and bonding features. For β-Si_3_N_4_, significant anisotropy along different crystal directions in thermal transportation is observed^6^, resulting in difficulty in achieving high thermal conductivity in polycrystalline ceramics with randomly distributed grains. Unlike β-Si_3_N_4_, the thermal conductivity of these two cubic phases is isotropic, which implies that preferred orientation of microstructure has no impact on the heat conduction. This is a great retrenchment in complexity and cost in the preparation and shaping of ceramic substrates. Moreover, no anisotropy of thermal expansion, which is responsible for the thermal-cycling induced cracking, exists in cubic phases, and the thermal stress from mismatched thermal expansion between substrate and silicon can be minimized since γ-Si_3_N_4_ matches well with silicon in thermal expansion^22^.

In summary, the lattice dynamics and thermal conductivity of two promising substrate materials, γ-Si_3_N_4_ and γ-Ge_3_N_4_, have been investigated through the combination of density functional theory and modified Debye-Callaway model. The high thermal conductivities of these two nitrides are verified, *κ* at RT is higher than 250 W∙m^−1^∙K^−1^ for samples with grain size of 100 μm for both compounds. High thermal conductivity is contributed to relatively small anharmonicity, large acoustic phonon velocities and Debye temperatures. Besides high RT κ, isotropy in thermal conductivity and expansion also promise γ-Si_3_N_4_ and γ-Ge_3_N_4_ a bright perspective as substrate material for high-power electronic devices by reducing the thermal stress and cracking during service.

## Methods

### Intrinsic lattice thermal conductivity

γ-Si_3_N_4_ is an insulator with a band gap of 4.8 eV such that the main thermal conductivity arises from its lattice contributions. The temperature-dependent lattice thermal conductivity (*κ*_L_) can be calculated by the approach of modified Debye-Callaway model^27^. In the formulism of this model, the total *κ*_L_ is the sum of contributions from two transverse (${\kappa }_{TA}$ and ${\kappa }_{TA^{\prime} }$) and one longitudinal (${\kappa }_{LA}$) acoustic phonon branches without considering the contributions of optical phonons due to their low group velocity^28^:

2 $${\kappa }_{T}={\kappa }_{LA}+{\kappa }_{TA}+{\kappa }_{TA\text{'}}$$

The partial conductivities *κ*_i_ (*i* represents LA, TA, and TA′ phonon branches) are the usual Debye-Callaway terms given by:

3 $${\kappa }_{i}=\frac{1}{3}\frac{{k}_{B}^{4}{T}^{3}}{2{\pi }^{2}{\hslash }^{3}{v}_{i}}{\int }_{0}^{{\theta }_{i}/T}\frac{{\tau }_{C}^{i}(x){x}^{4}{e}^{x}}{{({e}^{x}-1)}^{2}}dx$$

where *k*_B_ is the Boltzmann constant, $\hslash$ is the reduced Planck constant, *v*_i_ is the phonon group velocity, *τ*_C_ is the total relaxation time of phonon scattering process, $x=\hslash \omega /{k}_{B}T$, *θ*_i_ is the longitudinal and transverse acoustic Debye temperature which is estimated by ${\theta }_{i}=\hslash {\omega }_{\max }/{k}_{B}$, where *w*_max_ is the maximum frequency of the acoustic phonon branch at the Brillouin zone (BZ) boundary^29,30^.

The total resistive phonon scattering rate (*τ*_C_^−1^) for a crystal is the sum of scattering rates due to phonon-phonon Umklapp scattering (*τ*_U_^−1^), isotope scattering (*τ*_I_^−1^) and boundary scattering (*τ*_B_^−1^)^27^:

4 $${\tau }_{C}^{-1}={\tau }_{U}^{-1}+{\tau }_{I}^{-1}+{\tau }_{B}^{-1}$$

For a perfect insulator, the Umklapp scattering dominate the phonon scattering process at high temperatures, which leads to *τ*_C_ ≈ *τ*_U_. While at low temperatures, the main phonon scattering process is the isotope and boundary effect^27^. In order to estimate the upper limit of *κ*_L_, in this work, we only considered the effect of grain size (*τ*_B_) on the thermal conductivity, whereas the effects of isotope and defects are ignored. Slack *et al*. suggested the following expression to estimate the relaxation time of Umklapp scattering process^31^:

5 $${[{\tau }_{U}^{i}]}^{-1}=\frac{\hslash {\gamma }_{i}^{2}}{M{v}_{i}^{2}{\theta }_{i}}{(\frac{{k}_{B}}{\hslash })}^{2}{x}^{2}{T}^{3}\exp (-\,{\theta }_{i}/3T)$$

where *M* is the average mass of an atom in the crystal, γ_i_ is the Grüneisen parameter of the acoustic phonon branch *i*, which is calculated from the mode Grüneisen parameter weighed by mode specifc heat capacity. The mode Grüneisen parameter for the mode *i* at the wave vector ***q*** is defined by:

6 $${\gamma }_{i,q}=-\frac{\partial \,\mathrm{ln}\,{\omega }_{i,q}}{\partial \,\mathrm{ln}\,V}$$

where *V* is the volume of the unit cell. Then, the Grüneisen parameter for different acoustic phonon is estimated by^32^:

7 $${\gamma }_{i}=\frac{\sum {\gamma }_{i,q}{C}_{i}(q)}{\sum {C}_{i}(q)}$$

8 $${C}_{i}(q)={k}_{B}{(\frac{\hslash {\omega }_{i,q}}{{k}_{B}T})}^{2}\frac{\exp (\hslash {\omega }_{i,q}/{k}_{B}T)}{{[\exp (\hslash {\omega }_{i,q}/{k}_{B}T)-1]}^{2}}$$

The phonon-boundary scattering rate is independent of temperature and frequency, and can be written as^27^:

9 $${[{\tau }_{B}^{i}]}^{-1}=\frac{{v}_{i}}{d}$$

where *d* is the effective diameter of the grain size in samples.

### First-principles calculations

Density functional theory (DFT) calculations were performed using the Cambridge Serial Total Energy Package (CASTEP) code^33^. The ultra-soft pseudopotentials were employed to represent the interactions between the ionic cores and the valence electrons^34^. The exchange-correlation energy was treated under the generalized-gradient approximation (GGA)^35^. The plane-wave basis set cut-off energy was fixed at 350 eV after convergence tests. The special points sampling integration over BZ was conducted by Monkhorst-Pack method with a 7 × 7 × 7 special *k*-points mesh^36^. The geometry optimization was achieved under the Broyden-Fletcher-Goldfarb-Shanno (BFGS) minimization scheme^37^. Lattice parameters and internal atomic positions are optimized until the total energy and maximum ionic displacement converge to 5 × 10^−6^ eV/atom and 5 × 10^−4^ Å, respectively.

Lattice-dynamics calculations are calculated within the framework of density function perturbation theory (DFPT) via finite displacement method^38^. The force constants matrix was calculated by perturbing the positions of atoms slightly in a 2 × 2 × 2 supercell, and the phonon frequencies and dispersion were obtained by solving the dynamical matrices. A 7 × 7 × 7 *k*-points mesh was used in the calculation of force constants matrix.

## Data Availability

All data generated or analysed during this study are included in this published article.
